# Functional Metagenomics of the Bronchial Microbiome in COPD

**DOI:** 10.1371/journal.pone.0144448

**Published:** 2015-12-03

**Authors:** Laura Millares, Vicente Pérez-Brocal, Rafaela Ferrari, Miguel Gallego, Xavier Pomares, Marian García-Núñez, Concepción Montón, Silvia Capilla, Eduard Monsó, Andrés Moya

**Affiliations:** 1 Fundació Parc Taulí, Sabadell, Spain; 2 CIBER de Enfermedades Respiratorias, CIBERES, Bunyola, Spain; 3 Universitat Autònoma de Barcelona, Esfera UAB, Barcelona, Spain; 4 Fundació Insitut d’Investigació Germans Trias i Pujol, Badalona, Spain; 5 Genomics and Health Area, Fundación para el Fomento de la Investigación Sanitaria y Biomédica de la Comunidad Valenciana (FISABIO-Public Health), Valencia, Spain; 6 CIBER Epidemiología y Salud Pública (CIBERESP), Barcelona, Spain; 7 Evolutionary Genetics Unit, Institut Cavanilles de Biodiversitat i Biologia Evolutiva (ICBiBE), Universitat de València, Valencia, Spain; 8 Department of Respiratory Medicine, Hospital Universitari Parc Taulí, Sabadell, Spain; 9 Department of Microbiology, Hospital Universitari Parc Taulí, Sabadell, Spain; Queens University Belfast, IRELAND

## Abstract

The course of chronic obstructive pulmonary disease (COPD) is frequently aggravated by exacerbations, and changes in the composition and activity of the microbiome may be implicated in their appearance. The aim of this study was to analyse the composition and the gene content of the microbial community in bronchial secretions of COPD patients in both stability and exacerbation. Taxonomic data were obtained by 16S rRNA gene amplification and pyrosequencing, and metabolic information through shotgun metagenomics, using the Metagenomics RAST server (MG-RAST), and the PICRUSt (Phylogenetic Investigation of Communities by Reconstruction of Unobserved States) programme, which predict metagenomes from 16S data. Eight severe COPD patients provided good quality sputum samples, and no significant differences in the relative abundance of any phyla and genera were found between stability and exacerbation. Bacterial biodiversity (Chao1 and Shannon indexes) did not show statistical differences and beta-diversity analysis (Bray-Curtis dissimilarity index) showed a similar microbial composition in the two clinical situations. Four functional categories showed statistically significant differences with MG-RAST at KEGG level 2: in exacerbation, *Cell growth and Death* and *Transport and Catabolism* decreased in abundance [1.6 (0.2–2.3) *vs* 3.6 (3.3–6.9), p = 0.012; and 1.8 (0–3.3) *vs* 3.6 (1.8–5.1), p = 0.025 respectively], while *Cancer* and *Carbohydrate Metabolism* increased [0.8 (0–1.5) *vs* 0 (0–0.5), p = 0.043; and 7 (6.4–9) *vs* 5.9 (6.3–6.1), p = 0.012 respectively]. In conclusion, the bronchial microbiome as a whole is not significantly modified when exacerbation symptoms appear in severe COPD patients, but its functional metabolic capabilities show significant changes in several pathways.

## Introduction

The course of severe chronic obstructive pulmonary disease (COPD) is often impaired by exacerbations which are characterized by a sustained worsening of respiratory symptoms over the daily variability of the disease [1]. Culture-based procedures diagnose bacterial infection in ~50% of these episodes [2], *Haemophilus influenzae*, *Streptococcus pneumoniae*, *Moraxella catarrhalis* and *Pseudomonas aeruginosa* being the most commonly identified pathogens [3]. Molecular culture-independent techniques have recently broadened our knowledge of the bacterial communities in the bronchial tree of COPD patients, both when the disease is stable and during exacerbations [4–6], but the role of most bacteria identified by these methods remains unknown, as do their interactions with the bronchial tree [4]. Recent studies have suggested that exacerbation patterns in COPD may be related to the bacterial microbiota as a whole and not just to a narrow range of well-known pathogenic bacteria, which are currently considered to be the cause of most of these acute episodes [4,7].

Changes in the composition of the bacterial community can be identified through 16S ribosomal RNA gene sequencing, and an overgrowth of specific pathogenic bacteria has been described in exacerbations of severe COPD patients [6]. However, this analysis provides no information on the metabolic activity and function of the bronchial microbiota [8], whose characterization may be approached by other techniques such as shotgun metagenomics. Millions of fragments of short DNA reads are created when this approach is used, and after de-replication and quality control, the fragments obtained may be mapped to databases of orthologous gene groups such as KEGG (Kyoto Encyclopedia of Genes and Genomes) [9] to identify matches to genes or proteins with previously described functions [10,11]. However, this approach depends on the isolation of sufficient quantities of bacterial DNA, and other method has recently been developed to investigate the functional profiles of the microbiota. PICRUSt (Phylogenetic Investigation of Communities by Reconstruction of Unobserved States) uses evolutionary modelling to predict metagenomes from 16S data and a reference genome database [12]. Though this approach has limitations, such as the fact that the software does not distinguish differences at strain level and cannot detect genes not included in the genomic database used [12,13], it is useful for detecting microbial function and its variability, when the quantity of bacterial DNA present is low [8].

The aim of this study is to analyse the gene content of the microbial community in COPD in both stability and exacerbation, in order to identify the functional changes in the bronchial microbiota which are associated with the appearance of an acute impairment. To do so, 16S rRNA was first amplified and pyrosequenced to determine the taxonomy of the bronchial microbiota in severe COPD patients, and subsequently, the metabolic information of the microbial community was assessed through PICRUSt. Finally, microbial function was assessed by shotgun metagenomics using the Metagenomics RAST server (MG-RAST) [14].

## Methods

### Ethics Statement

Ethical permission for the study was obtained from the Sabadell Hospital Ethics Committee and a written informed consent was obtained from each subject at enrolment.

### Design and population

Outpatients from a severe COPD cohort regularly attending a Day Care Unit for scheduled and exacerbation visits [6,15] were selected for this study. The cohort included COPD patients with a FEV_1_ below 50% from the reference [16], who reported three or more exacerbations in the previous year and who had attended the Day Care Unit of Sabadell University Hospital since 2005. Patients in the cohort had scheduled visits every three months from their inclusion and unscheduled visits when exacerbations symptoms appeared, as described elsewhere [15]. Previous diagnosis of asthma, cystic fibrosis, bronchiectasis, cancer and chronic treatment with immunosuppressive drugs were exclusion criteria [6,17]. For the purposes of the study, patients were followed for one year after their baseline assessment, with follow-up visits which included sputum sampling and culture scheduled every three months, until the first unscheduled exacerbation visit. As previously described [6] acute episodes of increased dyspnoea, sputum production and/or purulence appearing during follow-up and treated with antibiotics and/or oral corticosteroids were considered as exacerbations [18,19]. Patients in the cohort were included in the study when they provided good quality sputum samples for culture from an exacerbation and from a previous stability visit 1–6 months before the acute episode. Participants should have not taken antibiotics between both samplings, and the amount of DNA extracted had to be sufficient for the proposed analyses [6].

### Clinical variables and sputum collection

Spontaneous sputum was collected from each patient in clinical stability, and during the subsequent exacerbation before the administration of antibiotic therapy. Functional characteristics were assessed at baseline and included forced spirometry, reversibility testing [20] and BODE index [21] as previously described [6]. Microbiological processing for potential pathogenic microorganisms (PPM) detection by culture followed local clinical culture guidelines, and is reported elsewhere in detail [6].

### DNA extraction

DNA extraction was performed according to previously detailed procedures [6,22]. Briefly, sputum samples diluted with dithiothreitol were incubated at 37°C for 15 minutes and centrifuged for 10 minutes at 4°C. The pellet was resuspended in 1 ml of an in-house lysis buffer consisting of 100 U/mL mutanolysin, 47,700 U mL lysozyme and 2 U/mL lysostaphin dissolved in autoclave-sterilized MiliQ water. DNA was extracted with QIAamp DNA Blood Midi kit (Qiagen, Helden, Germany) in a Class II Biological Safety Cabinet to avoid external contamination. DNA was quantified in the nanodrop ND-1000 Spectrophotometer (NanoDrop Tecnologies, Inc., Wilmington, USA) and stored at -80°C for further determinations.

### PCR amplification of the V1-V3 region of the 16S rRNA gene

The hypervariable regions V1, V2 and V3 of the 16S rRNA gene were amplified following a previously described methodology [6]. PCR conditions were 5 min of initial denaturation at 94°C followed by 25 cycles of denaturation (30 s at 94°C), annealing (30 s at 52°C) and elongation (1 min at 72°C). After amplification, the products were visualized in 2% agarose gels. In order to assess the absence of contaminant DNA in the extraction buffer, controls were PCR amplified in parallel with the samples, and no bands were detected in the gel electrophoresis. Amplified products were purified with NucleoFast 96 PCR Clean-Up kit (Macherey-Nagel, GmbH & Co. KG, Germany), eluted in 28 μl of PCR-grade water and quantified using QuantiT PicoGreen dsDNA Assay Kit (Invitrogen, Life technologies, Carlsbad, USA). Sixteen samples with different barcode sequences were pooled in equimolar amounts into a single tube and pyrosequencing was carried out using the Roche 454 GS-FLX System Titanium Chemistry (Roche, Switzerland).

### Sequence analysis and microbiome accession numbers

16S rRNA raw sequences were analysed with the MOTHUR software package 1.27 [23]. The sequences with low quality scores (<20) and read lengths <200 and >520bp were removed [6]. Thereafter, the remaining sequences were aligned and checked for potential chimeras applying the align.seqs and chimera.slayer tools incorporated in MOTHUR. The Quantitative insights into microbial ecology (QIIME) pipeline [24] (MacQIIME 1.8.0) was used for sequence processing to obtain taxonomic information using Greengenes13_8 sequence database [25] as reference and RDP classifier 2.2 [26]. *De novo* operational taxonomic units (OTUs) picking method was used with UCLUST [27] and PyNAST version 1.2.2 as alignment method [28]. Bacterial 16S rRNA data sets from this study are accessible in the European Nucleotide Archive under the study accession number PRJEB4144, available at the URL http://www.ebi.ac.uk/ena/data/view/PRJEB4144, with the sample accession numbers ERS255717, 20,21,23–26,29–33,36–39.

### Shotgun pyrosequencing

The DNA concentration from all samples was measured by PicoGreen fluorescence in a Modulus 9200 fluorimeter from Turner Biosystems (Sunnyvale, USA).The pyrosequencing library was performed following standard procedures with GS-FLX Titanium Rapid Library kit (Roche, Switzerland). Briefly, DNA was fragmented by nebulization and after fragment end repair and purification, adaptors were added by ligation and small fragments were removed with Agencourt AMPure XP kit (Beckman Coulter, USA). Finally, the library was quantitated and prepared for emulsion PCR. The 16 samples with different tags were mixed in equimolar amounts and directly sequenced at the Foundation for the Promotion of Sanitary and Biomedical Research (FISABIO, Valencia, Spain), with the Roche 454 GS-FLX System Titanium Chemistry, using a full sequencing plate.

### Functional analysis

The PICRUSt software package was used for the predictive functional analysis. This software estimates the community metagenome using 16S rRNA sequencing data. KEEG (Kyoto Encyclopedia of Genes and Genomes) pathway was used to identify metagenomic contents. For shotgun pyrosequenced data, sequences were first analysed with the MOTHUR software package 1.27 to remove sequences shorter than 100 bp. Then, reads were aligned with the Cluster Database at High Identity (cdhit-454) with a sequence identity cutoff of 1 [29]. The sequences were compared against the Small Subunit rRNA Reference Database (SSUrdb) with an e-value cutoff of 1e^-10^ and all sequences that remained unassigned as SSU rRNA were analysed with the Large Subunit rRNA Reference Database (LSUrdb) with an e-value cutoff of 1e^-4^ as described by Urich et al [30]. The remaining sequences were analysed with the MG-RAST pipeline version 3.3 [14]. MG-RAST is a data repository, an analysis pipeline and a comparative genomics environment which provides quality control, feature prediction and functional annotation [11]. Quality control of the sequences was done by using the default parameters of MG-RAST in length, ambiguous base and dereplication filtering.The metabolic potential of the microbiota was examined using KEGG with default parameters, maximum e-value of 10^−5^, minimum identity of 60%, and minimum alignment length of 15. Abundance profiles of functional annotations were obtained by both methods, and differences in the functional genomic content were evaluated after normalizing the abundances of each category to the total number of proteins predicted for each sample. Shotgun sequences are accessible in the MG-RAST server (http://metagenomics.anl.gov/linkin.cgi?project=3296), with the following accession numbers: 4512860.3, 4512862.3, 4512863.3, 4512865.3–67.3, 4512869.3, 4512871.3–75.3, 4512877.3, 4512878.3, 4512880.3, 4512881.3.

### Statistical analyses

Statistical analyses were performed using the SPSS statistical software package version 18 (SPSS Inc., Chicago, IL, USA). Results for categorical variables are expressed as absolute and relative frequencies, and results for continuous variables as means and standard deviations (SD) or as medians and interquartile ranges (IQR) when the distribution was not normal [6]. Bacterial diversity was assessed through the Chao1 estimator [31] and the Shannon index [32], calculating both indexes after subsampling with QIIME to avoid sequencing effort bias [6]. Principal Coordinates Analysis (PCoA) and Bray-Curtis dissimilarity index [33] were used to study community composition, assessing the statistical significance of the differences in sample groupings through Bray-Curtis distance matrices and Adonis testing. Functional categories and their relative abundance in exacerbation samples were compared with the stability reference using Wilcoxon test for paired data, and correlations between PICRUSt and MG-RAST data were assessed using the Spearman's rank correlation coefficient. Statistical tests used in the study were two-sided, and a p value of 0.05 or less was reported as statistically significant [6].

## Results

### Patient characteristics and 16S rRNA analyses in stable COPD

The cohort consisted of 118 patients who provided good quality sputum samples for microbiologic analysis in the year after enrolment. Eight severe COPD patients met the criteria for inclusion in the study and provided good quality sputum samples from a stability period between one and six months before an exacerbation with sufficient quantities of bacterial DNA to perform the analyses scheduled.

PPMs were isolated from seven baseline stability samples (87.5%), with *Haemophilus influenzae* and *Pseudomonas aeruginosa* as the most frequently recovered colonizing microorganisms (Table 1).

**Table 1:** Baseline clinical characteristics.

N	8
Age (years), mean (SD)	72 (7)
Male, n (%)	8 (100)
Smoking (pack-year), median (IQR)	67 (32–110)
FEV_1_ post-BD (% predicted), mean (SD)	37 (8)
Inhaled corticosteroid treatment, n (%)	8 (100)
Positive cultures in stability	7 (87.5)
Microorganisms isolated	
- * Haemophilus influenzae*	3 (37.5)
- * Pseudomonas aeruginosa*	3 (37.5)
- * Moraxella catarrhalis*	2 (25)
- * Streptococcus pneumoniae*	1 (12.5)
- * Alcaligenes* spp.	1 (12.5)

SD, standard deviation; FEV_1_ post-BD, forced expiratory volume in one second post bronchodilatation.

Regarding culture-independent analysis, the most prevalent phyla in stability were *Proteobacteria* (52%), *Firmicutes* (26%) and *Actinobacteria* (19%) (Table 2). At genus level, 68 different OTUs were found: *Streptococcus* and *Haemophilus* were the most prevalent genera, accounting for over 50% of the observed relative abundance (S1 Table).

**Table 2:** Relative abundance of the phyla detected in stability and exacerbation samples.

Phylum	Relative abundance		
	Stability	Exacerbation	*p-*value
*Proteobacteria*	58 (24–83)	71 (13–89)	0.575
*Firmicutes*	15 (11–31)	21 (5–50)	0.779
*Actinobacteria*	7 (2–23)	7 (1–18)	0.779
*Bacteroidetes*	1 (0.3–2.5)	2 (0.1–3)	0.674
*Fusobacteria*	0.4 (0.3–1)	0.8 (0.1–1.5)	0.866
*TM7*	0 (0–0.04)	0 (0–0.5)	0.116
*Tenericutes*	0 (0–0.03)	0 (0–0.1)	0.237

### 16S rRNA analyses in exacerbated COPD

**Fig 1:**
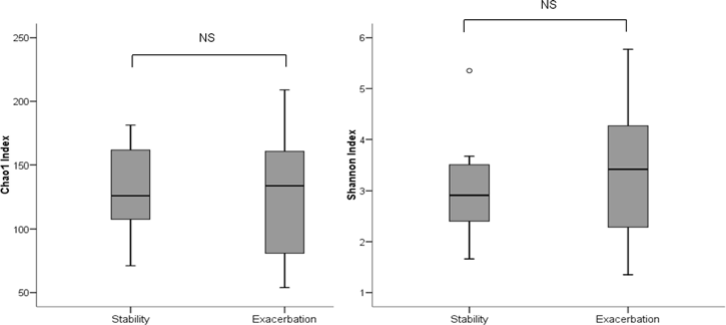
Diversity indexes in stability and exacerbation in severe COPD. A) Chao1 index and B) Shannon index. (Wilcoxon test).

Samples recovered from exacerbation gave positive cultures for PPMs in five of the eight patients studied (62.5%). *Haemophilus influenzae*, *Pseudomonas aeruginosa*, *Streptococcus pneumoniae* and *Staphylococcus aureus* were the microorganisms isolated. Regarding culture-independent analysis, no statistically significant differences were found in the relative abundances of the present bacterial microbiota between stability and exacerbation at either phylum or genus level (Table 2 and S1 Table). Bacterial diversity showed similar figures in the two clinical situations when measured through the Chao1 richness estimator [median (IQR); stability 134 (76–166) *vs* exacerbation 126 (101–165.5); p = 0.779, Wilcoxon test], and the Shannon index [3 (2–4) *vs* 3 (2–3.5); p = 0.575, Wilcoxon test] (Fig 1). Similarly, the assessment of the microbial composition using sample grouping at PCoA and the Bray-Curtis dissimilarity index did not show differences between stability and exacerbation, and Adonis testing confirmed that the microbial composition as a whole did not differ in the two clinical situations (R^2^ = 0.02, p = 0.955) (S1 Fig).

### Prediction with PICRUSt

**Fig 2:**
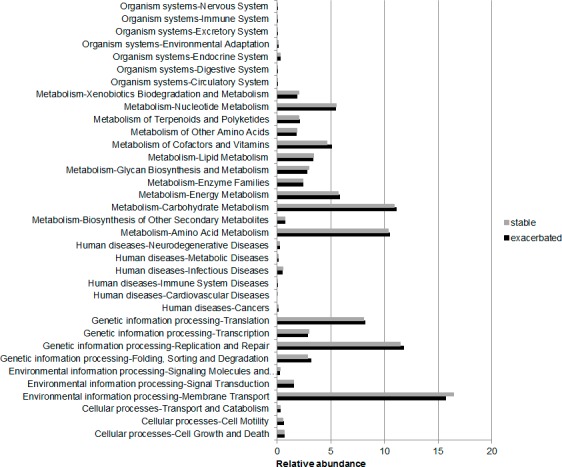
Functional categories obtained with PICRUSt for stability and exacerbation (KEGG database at level 2).

The PICRUSt programme was used to predict the functional capacities of the bacterial community through 16S sequences. The differences observed between stability and exacerbation did not reach statistical significance for any of the six functional categories defined at KEGG level 1 (*Cellular Processes*, *Environmental Information Processing*, *Genetic Information Processing*, *Human Diseases*, *Metabolism* and *Organism Systems*). Similar results were obtained at level 2 for the 35 functional categories observed in the studied group out of the 44 categories that form this level (Fig 2).

### Analysis with MG-RAST

**Fig 3:**
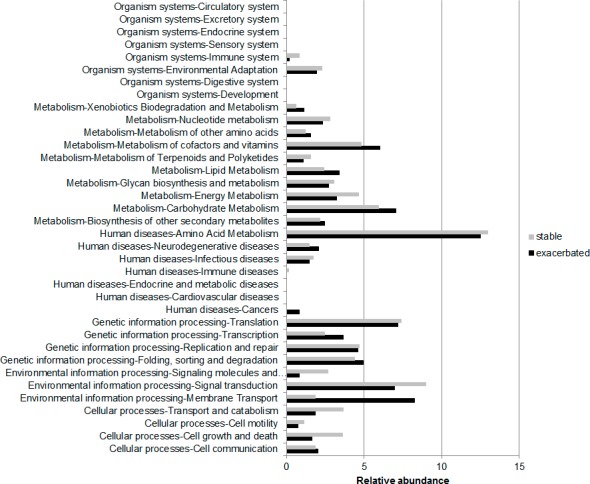
Functional categories obtained with MG-RAST for stability and exacerbation (KEGG database at level 2).

**Fig 4:**
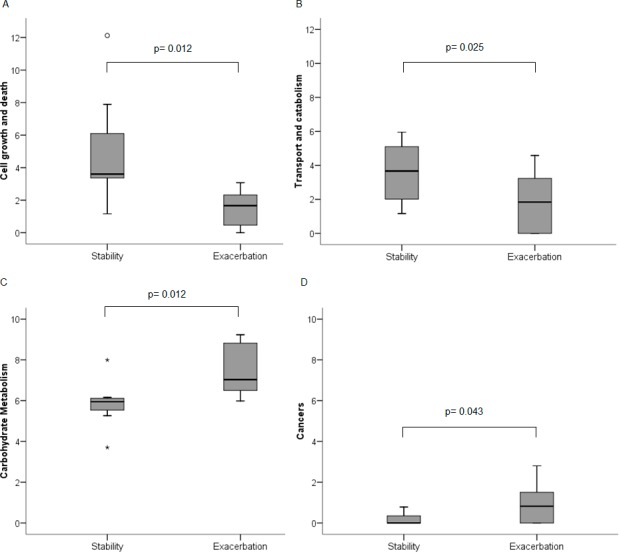
Functional categories with statistically significant differences between stability and exacerbation at level 2. A) Cell growth and dead pathway; B) Transport and catabolism pathway; C) Carbohydrate metabolism pathway and D) Cancer pathway.(Wilcoxon test).

A mean of 13,700 (SD 6,521) sequences per sample were uploaded to the MG-RAST server, with no differences between the number of available sequences from stability and exacerbation samples [mean (SD); 11,088 (3,488) *vs* 16,312 (7,960); p = 0.382; Mann-Whitney U test]. Functional information was obtained from the sequences that met the quality criteria (78%). Sequences belonging to prokaryotic organisms represented medians of 18% (12–30) in stability and 12.5% (9.5–22) in exacerbation samples (p = 0.279, Mann-Whitney U test). In exacerbation samples, higher abundance at level 1 was found for the *Metabolism* category [median (IQR); 40 (37–42) *vs* 35 (32–40); p = 0.012, Wilcoxon test]. At level 2, four out of the 36 categories observed showed statistically significant differences in exacerbation when compared with stability (Fig 3). *Carbohydrate Metabolism* and *Cancer* were significantly more abundant in exacerbations [7 (6.4–9) *vs* 5.9 (6.3–6.1); p = 0.012; and 0.8 (0–1.5) *vs* 0 (0–0.5); p = 0.043 respectively; Wilcoxon test]. In contrast, *Cell growth and death* and *Transport and Catabolism* categories showed lower abundance in exacerbation samples [1.6 (0.2–2.3) *vs* 3.6 (3.3–6.9); p = 0.012; and 1.8 (0–3.3) *vs* 3.6 (1.8–5.1); p = 0.02; Wilcoxon test] (Fig 4).

### Comparison of functional inferential techniques

As a secondary aim, we evaluated whether there was a correlation between the functional capacities of the microbiome predicted by PICRUSt and the results obtained by shotgun pyrosequencing. A significant positive correlation between relative abundances obtained with PICRUSt and MG-RAST at level 1 in KEGG database was found in both stability and exacerbation samples (rho = 0.886, p = 0.019 and rho = 1, p< 0.01; respectively). With PICRUSt, 35 functional categories were found at level 2, compared with 36 with MG-RAST, with only two categories differing between them. A significant positive correlation was also found between PICRUSt and MG-RAST relative abundances for these categories, in both clinical situations (rho = 0.734, p<0.01; and rho = 0.807, p<0.01; for stability and exacerbation respectively).

## Discussion

In this study we assessed the bacterial community structure in severe COPD patients by assessing 16S rRNA pyrosequencing and its metabolic functionality, using prediction through PICRUSt and shotgun metagenomics with MG-RAST. Sputum samples in stability and during a subsequent exacerbation were analysed in order to identify the changes both in the community composition and in its functional capacities, which may be associated with the appearance of acute impairments. Considering the group as a whole, no differences in the bronchial microbiota were found between stability and exacerbation samples, but clear functional differences were identified during exacerbations in the *Carbohydrate Metabolism*, *Cancer*, *Cell Growth and Death* and *Transport and Catabolism* categories in MG-RAST. PICRUSt prediction showed a good correlation with MG-RAST, but differences between the categories did not reach statistical significance when assessed using this predictor. These results suggest that the bronchial microbiota as a whole do not change significantly during COPD exacerbations, but the resident community modifies its metabolic functional patterns during exacerbations.

The microbiome composition did not show differences between the samples obtained in stability and exacerbation in our study, suggesting that most of the bacterial community do not suffer significant changes in their relative abundance during acute episodes in COPD patients. These results agree with previous observations that have identified that increases in relative abundance during exacerbations are restricted to some genera, which may account for the appearance of acute symptoms [6]. Similarly, analysing the changes in the bacteria composition in serial samples obtained before, during and after an exacerbation in COPD patients, Huang *et al*. [7] found that the microbial composition as a whole did not show large changes in exacerbations. Likewise, in other respiratory diseases such as cystic fibrosis and bronchiectasis, no differences in diversity and community composition were observed between samples obtained during stability and at the time of an exacerbation [34,35]. In our study, 16S rRNA analysis showed a high prevalence of two genera, *Streptococcus* and *Haemophilus*, in the bronchial microbiome of COPD patients, without statistical differences between stability and exacerbation. In other chronic respiratory diseases as cystic fibrosis and bronchiectasis, it has also been reported that a restricted number of taxa form the main part of the bronchial microbiota, while a wide range of taxa, which have low abundance in bronchial secretions, account for most of the bacterial community richness observed [34,36,37]. Overall, these observations suggest that the context of the microbial community as a whole may be important in order to understand the pathogenesis of COPD exacerbations.

Our results demonstrate that, in spite of the lack of differences in community composition between stability and exacerbation in severe COPD patients, significant differences in bacterial metabolic functionalities may appear, especially in the *Cancer*, *Cell Growth and Death*, *Carbohydrate Metabolism and Transport* and *Catabolism* pathways. Although metagenomic analysis is not able to provide information on the end product expression profile of bacterial communities [38], it accurately describes the genomic potential of the community, which is reflected in the genes encoded into their genomes [8]. The PICRUSt programme may be used to predict the genomic content of the bacterial community through 16S phylogenetic information, which has been shown to be well correlated with the genomic content [12]. Although the use of this 16S-based phylogeny to infer function is cost-effective, it offers only an estimation [13]. Deep metagenomic sequencing, as well as metatranscriptomics, metabolomics and metaproteomics are required to characterize more accurately the microbiome function [12,13]. In our study, in agreement with previous results [12], the two techniques were correlated, but statistically significant results were only attained through MG-RAST, confirming the importance of this approach. The relationship between phylogeny and gene content is not perfect [39], and may be the cause of the lower strength in the PICRUSt analyses.

Our results support the hypothesis that the functional capabilities of the microbiome change when an exacerbation appears, in a context of stability of the microbiota composition. In exacerbated COPD patients, Huang *et al* [7] also reported increases in various metabolic pathways involving viral and bacterial infection and apoptosis, and decreases in pathways associated with flavonoid and steroid biosynthesis and betalain and indole alkaloid production. Bacteria, then, have the ability to modify their functional pathways when the clinical situation of their host changes, without major modifications in the microbiota as a whole. From the results of our study it is not possible to determine whether the changes observed in the microbial function contribute to the appearance of symptoms or are only a consequence of the acute episodes. Similar changes in the functional capabilities of the regional microbiome have been found in patients with type 2 diabetes, who showed minimal alterations in their gut community composition but significant changes in functional annotations compared with healthy subjects, suggesting that functional alterations rather than changes in the abundance of specific microorganisms could be associated with the disease [40]. Likewise, in a study of patients suffering from hepatitis B liver cirrhosis, functional diversity was found to be significantly reduced in the fecal microbiota compared with healthy subjects [41].

The *Carbohydrate Metabolism* and *Cancer* pathways were enriched when an exacerbation appeared, while *Cell Growth and Death* and *Transport and Catabolism* decreased significantly in our study. The modification in the carbohydrate metabolism pathway in exacerbation is probably due to the fact that the main energy sources for bacteria were carbohydrates, and may be related to the decrease found in the *Transport and Catabolism* pathway. Carbohydrates have the ability to minimize catabolism through carbohydrate-mediated catabolite repression [42], which has been shown to modulate virulence gene expression in many microorganisms [43–46] such as the genus *Streptococcus*. It does so through three major mechanisms: acquisition of crucial nutrients, adherence to eukaryotic cells and interference with the function of host immunity proteins [45]. Overall, these changes in the functions of the bronchial microbiome may indicate higher virulence and pathogenic capacity of bacteria in exacerbations.

In the epidemiologic context of an increase in the incidence of lung cancer in COPD patients demonstrated in different population-based studies [47–49], the finding that the *Cancer* and *Cell Growth and Death* pathways change during exacerbations in severe COPD patients, who frequently present these episodes during their disease, may have clinical implications. Longitudinal studies would be needed to increase the insights into the relationships between the functionality of the bronchial microbiome and carcinogenesis in COPD.

The present study has some limitations that should be taken into account. First of all, the sample size was limited to eight COPD patients due to the use of strict selection criteria. Secondly, enrolled patients showed severely impaired lung function, and the results may not be extrapolable to patients with moderate COPD, in view of the differences in the bronchial microbiome previously described in these two groups of patients [22]. Thirdly, metagenomic shotgun sequencing is expensive and computationally intensive, which limits the number of samples analysed and the possibility of extrapolating the results obtained in these samples. Finally, quality control of the analyses performed is needed, because shotgun pyrosequencing is more difficult in samples which have low bacterial DNA content or contain mainly host DNA [12], as happens in sputum. In addition, low proportions of bacterial DNA may induce an underestimation of the metabolic contents.

In conclusion, the bronchial microbiome as a whole is not significantly modified by the appearance of exacerbation symptoms in severe COPD patients. However, the functional metabolic capabilities of the microbiome change significantly in several pathways, including *Cancer*, *Cell Growth and Death*, *Carbohydrate Metabolism* and *Transport and Catabolism*. This pattern of changes may have clinical implications for the natural history of COPD patients suffering from recurrent exacerbations.

## Supporting Information

S1 Fig Principal coordinate analysis (PCoA) with Bray-Curtis dissimilarity index. Blue dots: stability samples; Red dots: exacerbation samples.
(TIF)

S1 Table Genera detected in stability and exacerbation samples through culture-independent analysis. (Wilcoxon test).
(XLSX)
